# Mutated Von Hippel-Lindau-renal cell carcinoma (RCC) promotes patients specific natural killer (NK) cytotoxicity

**DOI:** 10.1186/s13046-018-0952-7

**Published:** 2018-12-04

**Authors:** Anna Maria Trotta, Sara Santagata, Serena Zanotta, Crescenzo D’Alterio, Maria Napolitano, Giuseppina Rea, Rosa Camerlingo, Fabio Esposito, Elvira Lamantia, Annamaria Anniciello, Giovanni Botti, Nicola Longo, Gerardo Botti, Sandro Pignata, Sisto Perdonà, Stefania Scala

**Affiliations:** 1 Functional Genomics, Istituto Nazionale per lo Studio e la Cura dei Tumori, Fondazione “G. Pascale”-IRCCS, Via Semmola, 80131 Naples, Italy; 2 Hematology-Oncology and Stem-Cell Transplantation Unit, Istituto Nazionale per lo Studio e la Cura dei Tumori, Fondazione “G. Pascale”-IRCCS, Naples, Italy; 3 Pathology Unit, Istituto Nazionale per lo Studio e la Cura dei Tumori, Fondazione “G. Pascale”-IRCCS, Naples, Italy; 4 Uro-Gynecological Department, Istituto Nazionale per lo Studio e la Cura dei Tumori, Fondazione “G. Pascale”-IRCCS, Naples, Italy; 5 Cell Biology and Biotherapy, Istituto Nazionale per lo Studio e la Cura dei Tumori, Fondazione “G. Pascale”-IRCCS, Naples, Italy; 6 Urology Division, University Federico II, Naples, Italy; 7 Department of Biochemistry, Biophysics and General Pathology, University of Campania “L. Vanvitelli”, Naples, Italy

**Keywords:** Von Hippel-Lindau, Natural killer, Renal cell carcinoma, CD107a, Tumor microenvironment

## Abstract

**Background:**

Previous evidence demonstrated that restoration of wild type VHL in human renal cancer cells decreased in vitro NK susceptibility. To investigate on the role of tumoral VHL status versus NK capability in renal cancer patients, 51 RCC patients were characterized for VHL mutational status and NK function.

**Methods:**

VHL mutational status was determined by direct DNA sequencing on tumor tissue. NK cytotoxicity was measured against specific target cells K562, VHL-wild type (CAKI-1) and VHL-mutated (A498) human renal cancer cells through externalization of CD107a and IFN-γ production. Activating NK receptors, NKp30, NKp44, NKp46, NKG2D, DNAM-1, NCAM-1 and FcγRIIIa were evaluated through quantitative RT-PCR. RCC tumoral Tregs were characterized as CD4^+^CD25^+^CD127^low^Foxp3^+^ and Treg function was evaluated as inhibition of T-effector proliferation.

**Results:**

VHL mutations were detected in 26/55 (47%) RCC patients. IL-2 activated whole-blood samples (28 VHL-WT-RCC and 23 VHL-MUT-RCC) were evaluated for NK cytotoxicity toward human renal cancer cells A498, VHL-MUT and CAKI-1, VHL-WT. Efficient NK degranulation and increase in IFN-γ production was detected when IL-2 activated whole-blood from VHL-MUT-RCC patients were tested toward A498 as compared to CAKI-1 cells (CD107a^+^NK: 7 ± 2% vs 1 ± 0.41%, *p* = 0.015; IFN-γ^+^NK: 6.26 ± 3.4% vs 1.78 ± 0.9% respectively). In addition, IL-2 activated NKs induced higher CD107a exposure in the presence of RCC autologous tumor cells or A498 as compared to SN12C (average CD107a^+^NK: 4.7 and 2.7% vs 0.3% respectively at 10E:1 T ratio).

VHL-MUT-RCC tumors were NKp46^+^ cells infiltrated and expressed high NKp30 and NKp46 receptors as compared to VHL-WT-RCC tumors. A significant lower number of Tregs was detected in the tumor microenvironment of 13 VHL-MUT-RCC as compared to 13 VHL-WT-RCC tumors (1.84 ± 0.36% vs 3.79 ± 0.74% respectively, *p* = 0.04). Tregs isolated from VHL-MUT-RCC patients were less suppressive of patients T effector proliferation compared to Tregs from VHL-WT-RCC patients (Teff proliferation: 6.7 ± 3.9% vs 2.8 ± 1.1%).

**Conclusions:**

VHL tumoral mutations improve NKs effectiveness in RCC patients and need to be considered in the evaluation of immune response. Moreover therapeutic strategies designed to target NK cells could be beneficial in VHL-mutated-RCCs alone or in association with immune checkpoints inhibitors.

**Electronic supplementary material:**

The online version of this article (10.1186/s13046-018-0952-7) contains supplementary material, which is available to authorized users.

## Background

Renal cell carcinoma (RCC) is the most common kidney tumor in adults. Annually ~ 295,000 new kidney cancer cases are diagnosed and ~ 134,000 deaths are recorded worldwide [1, 2]. The most common histotype is clear cell-RCC (ccRCC) (70–80%) of which 50–70% carry Von Hippel-Lindau (VHL) gene mutations (VHL promoter hyper methylation and biallelic VHL inactivation) [3–6]. The VHL protein (pVHL) functions as an E3 ubiquitin ligase that targets the hypoxia-inducible factors (HIFs) for ubiquitin-mediated degradation via the proteasome [7–9]. Although VHL plays a key role in RCC pathogenesis, the prognostic meaning of VHL mutations is controversial. VHL mutations were reported not to affect prognosis [10, 11] or to improving it [12]. Natural killer cells (NKs) are effectors of the innate immune response [13] activated by a net between inhibitory and activating receptors: human activating receptors comprise the natural cytotoxicity receptors (NCRs: NKp46, NKp30, and NKp44), C-type lectin receptors (CD94/NKG2C, NKG2D, NKG2E/H, and NKG2F), and killer cell immunoglobulin-like receptors (KIRs) (KIR-2DS and KIR-3DS). Inhibitory receptors are C-type lectin receptors (CLRs) and KIRs (KIR-2DL and KIR-3DL) [14]. Certain VHL mutations correlate with low expression of classical HLA-I molecules and HLA-E expression that determine higher NK susceptibility reducing the engagement of KIR and NKG2A inhibitory receptors, respectively. Nevertheless multiple mechanisms concur in determining the NK sensitivity of cancer cells such as NK receptors surface ligands, LFA-1/ICAM-1 and HLA-I/NKR interactions, HLA-E molecules, soluble MICA A-B ligands, HLA-G, TGF beta secretion [15]. Moreover mechanisms affecting NK sensitivity may regulate gene transcription such as autophagy genes [16].

T regulatory cells (Tregs) partecipate in the control of tumor immunity attenuating antitumor immune response [17]. Increase in peripheral and tumoral Tregs were observed in several cancers and inversely correlated to NKs number and function [18]. Tregs mainly regulate NK activity in a transforming growth factor-beta (TGF-β)-dependent manner. Soluble and membrane-bound TGF-β on regulatory T cells impaired NK cytolytic activity through down-regulation of NKG2D receptor on the NK cells [19].

To investigate on the role of tumoral VHL status versus NK capability in renal cancer patients, 51 RCC patients were characterized for VHL mutational status and NK cytolytic activity toward A498-VHL-MUT and CAKI-1-VHL-WT human renal cancer cells.

## Materials and methods

### Patients and samples

Fifty five patients with primary RCC (41 clear cell; 4 papillary; 5 cromophobe; 5 unknown) and twelve healthy donors (HD) were enrolled in the study. The patients underwent surgery as part of their standard treatment at the Department of Urology, National Cancer Institute “G. Pascale” (Naples, Italy) and Genitourinary Oncology and Rare Cancer Center, Federico II University (Naples, Italy). RCC tumor and peritumoral tissues were obtained immediately after surgical resection, stabilized in RNA later (Qiagen) and stored at − 80 °C. The distance of 1 cm was the minimal distance between tumor and normal*-*appearing renal tissue sampled. Informed consent from each patient was sought. The research protocol was approved by Human Ethical Committee of Institute (n. CEI/423/13).

### Cell culture

The human renal cancer cell lines CAKI-1 (HLA-A*23:01/A*24:02; HLA-B*35:02/B*44:03; HLA-C*04/C*04new) and SN12C (HLA-A*03/A*24new; HLA-B*07/B*44; HLA-C*05/C*07:02) VHL wild-type (VHL-WT), A498 (HLA-A*02:01; HLA-B*08:01; HLA-C*07) and 786-O (HLA-A*03:01; HLA-B*07/B*44, HLA-C*05/C*07:02) VHL mutated (VHL-MUT) and the human leukemic cell line K562 were obtained from the NCI 60 cancer cell line collection [20, 21]. All cells were cultured in the recommended growth medium supplemented with 10% heat-inactivated fetal bovine serum (FBS), 1% L-glutamine, 1% penicillin/streptomycin and maintained in 95% air 5% CO^2^ at 37 °C.

### VHL somatic mutational status

VHL mutational status was determined by PCR amplification and direct sequencing. DNA was extracted from primary tumor tissues using Nucleon Genomic DNA extraction Kit (GE Healthcare). The three VHL coding exons, with exon-intron junctions, were PCR amplified using the following primers: exon 1, 5′-agcgcgttcc atcctctac-3′ (forward) and 5′-agttccccgtctgcaaaat-3′ (reverse); exon 2, 5′-aggacggtcttgatctcctg-3′ (forward) and 5′-gcccaaagtgcttttgagac-3′ (reverse); exon 3, 5′-ttgttggcaaagcctcttgt-3′ (forward) and 5′-tgcccctaaacatcacaatg-3′ (reverse). The thermal cycler (Applied Biosystems® 2720 Thermal Cycler) was programmed to first incubate the sample for 10 min for 95 °C followed by 35 cycles consisting of 95 °C for 45 s, 56 °C (exon1 and 3) and 60 °C (exon 2) for 45 s with final extension for 7 min at 72 °C. Purified PCR products were then sequenced by using the Big Dye terminators version 3.1 cycle sequencing kit (Applied Biosystems, Courtaboeuf, France) and the 3130 Genetic Analyzer (Applied Biosystems).

### Flow cytometry

FITC-conjugated major histocompatibility complex (MHC) class I–specific antibody (IgG2a, W6/32, CBL139F, Cymbus Biotech, Hants, UK) and PE anti-human CD155/PVR (clone SKIL.4, Biolegend, Cat No 337609) were used for flow cytometry. FACSAria II Cell Sorter 8-colour flow cytometer with BD FACSDiva™ 8.0 Software (BD Biosciences, San Jose, CA) was daily calibrated with calibrite beads (Fitc, Pe, PerCP and APC) and compbeads (Pe-Cy7 and APC-Cy7; BD Biosciences). PerCP-Cy5.5 anti-CD3 (BD Biosciences Cat No 560835), FITC-conjugated anti-CD56 (BD Biosciences, Cat No 345811) and PE-conjugate anti-CD107a antibody (BD Bioscience, Cat No 555801) were used to identify degranulating NK-cells from peripheral blood. For intracellular staining PE-conjugated anti-IFN-γ antibody (BD Biosciences Cat No 554701) was used. Fluorochrome-labelled monoclonal antibodies for identification of tissue Treg cells were: FITC-anti-FOXP3 (BD Biosciences, Cat No 560047), Pe-Cy7-anti-CD25 (BD Biosciences, Cat No 557741) and APC-Cy7-anti-CD4 (BD Biosciences, Cat No 557871). Intracellular staining for FoxP3 was performed using a commercially available kit (BD Cytofix/Cytoperm; fixation and permeabilization kit; BD Pharmingen) according to the manufacturer’s instructions. A minimum of 100.000 events for each sample were collected.

### NK cells and autologous tumor cells isolation

CD56^+^NK cells were isolated from PBMCs of 4 RCC patients (1 VHL-WT, 3 VHL-MUT) after Ficoll gradient centrifugation using the NK cell isolation kit (Miltenyi Biotec, Bergisch Gladbach, Germany). Purified NK cells were cultured in RPMI 1640 medium (GIBCO Invitrogen) in the presence of 100 U/ml recombinant interleukin 2 (IL-2) overnight at 37 °C, 5%CO_2_. Autologous tumor cells were isolated from tumor tissue with single cell sorting by FACS ARIAII post mechanical disruption and labelings with Antibody AbI RCC pure Mouse Dako (clone SPM314), 7AAD (Thermo Fischer) and PE anti-CD45 (BD Biosciences, cat No 555483) and AbII Goat anti-Mouse FITC IgG(H + L) (Caltag Laboratory) for indirect staining. Through the gate strategy 7AAD^−^/CD45^−^/RCC^+^ ~ 10,000 of tumor cells were isolate and cultured 24 h in 96-well plates with RPMI-1640 with 10% FBS medium.

### Tregs dependent-CSFE T effector proliferation assay

Tumor CD4^+^CD25^+^ T regulatory cells (Tregs) and peripheral CD4^+^CD25^−^ T effector (Teff) cells were isolated using the Dynabeads Regulatory CD4^+^CD25^+^ T cell kit as previously described [22]. All purification steps were performed according to the manufacturer’s instructions (Invitrogen by Life Technologies) and collected cells were found to be > 95% pure by flow cytometry. Tregs function was assessed by evaluating the CFSE-labeled Teff cells by FACS analysis. Carboxyfluorescein diacetate succinimidyl ester (CFSE)-labeled autologous CD4^+^CD25^−^ T cells from peripheral blood (CellTrace CFSE Cell Proliferation Kit, Molecular Probes, by Life Technologies) were cultured with tumor CD4^+^CD25^+^ Tregs in 1:1 ratio. Cells were cultured (5 × 10^3^ cells/well) in U-bottom 96-well plates with RPMI medium (Thermo scientific, HyClone Laboratories, Inc) supplemented with 2-mM L-glutamine, 100 U/ml penicillin, 100 μg streptomycin, and 10% fetal bovine serum. Cells were stimulated for 5 days in the presence of Dynabeads Human T-Activator CD3/CD28 (Gibco by Life Technologies).

### CD107a degranulation assay and IFN-γ production

Lysosome-associated membrane protein LAMP-1 (CD107a) is a marker of NK-cell degranulation. CD107a expression was measured by flow cytometry as previously described [23, 24]. Briefly, 1 ml whole blood was diluted with one volume RPMI-1640 containing 10% heat-inactivated fetal bovine serum and incubated with IL-2 (100 U/ml) overnight at 37 °C, 5% CO_2_. The cytotoxic activity of NK cells was tested against NK-sensitive cell line K562 (HLA class I-negative) [25], human renal cancer cell lines CAKI-1-VHL-WT, A498 and 786-OVHL-MUT. 200 μl of IL-2 activated blood was co-cultured with 2 × 10^5^ target cell lines in presence of PE-anti-CD107a antibody for 3-h at 37 °C in 5% CO_2_. Alternatively, purified IL-2 activated NK cells from peripheral blood of RCC patients were used as effector cells. The autologous RCC tumor cells, A498-VHL-MUT and SN12C-VHL-WT cell lines were used as target cells. Assays were performed at the indicated effector-target ratio (10E:1 T; 5E:1 T). To detect spontaneous degranulation samples were incubated in the absence of target cells. Following 3-h culture, cells were stained with anti-CD56 and NK cells were identified as CD3^−^CD56^+^ in the lymphocyte gate. CD107a was analyzed on CD56^+^CD3^−^ NK. For intracellular staining of IFN-γ, 200 μl of IL-2 preactivated blood samples were incubated with 2 × 10^5^ K562 or RCC target cells for 6 h at 37 °C in a humidified 5% CO_2_ incubator. The secretion inhibitor Brefeldin A (Sigma-Aldrich, Seelze, Germany) was added to the assay medium at a concentration of 10 μg/ml during the 6 h coincubation. After incubation, samples were placed on ice and stained with anti-CD56 and anti-CD3 mAbs, followed by erythrocyte lysis of blood samples with FACS Lysing Solution (BD Biosciences). Thereafter, cells were permeabilized using FACS permeabilizing solution (Perm2, BD Biosciences) and stained for intracellular IFN-γ with a PE-anti-IFN-γ antibody. Analysis was performed on a FACSAria II Cell Sorter 8-colour flow cytometer, blinded to patients’ clinical characteristics.

### Real-time PCR

RNA was isolated from tumoral and peritumoral RCC tissues using the RNeasy Mini kit (Qiagen, Hilden, Germany), according to manufacturer’s instructions. cDNA was synthesized from 1 μg of total RNA using SuperScript III Reverse Transciptase (Invitrogen). Quantitative real-time PCR was performed using SYBR Green PCR Master Mix (Applied Biosystems, Foster City, CA) and data were collected and quantitatively analyzed on an ABI Prism 7000 System (Applied Biosystems). Primers sequences for NCAM-1, FCGR3a (CD16), DNAM-1, NKG2D, NKp46, NKp44, NKp30,KLRB1 and TGFB1 are detailed in Additional file 1: Table S1. Relative mRNA expression was normalized with GUSB gene expression.

### Immunohistochemistry (IHC)

Paraffin-embedded RCC tissue blocks, after heat induced epitope retrieval with citrate buffer (pH 6), were incubated overnight at 4 °C with the mouse monoclonal IgG2B, anti-human NKp46 antibody, Clone 195,314 (MAB1850 R&D Systems) diluted 1:50, followed by incubation with peroxidase-conjugated secondary antibodies according to the manufacturer’s instructions (EnVision+System, Dako North America, Inc., Carpinteria, CA). The NKp46 receptor demonstrates a high degree of lineage-specificity, being expressed almost exclusively in natural killer cells._._ Five randomly selected areas were identified across the entire section from the center to the invasive margin (100× magnification). NKp46^+^ cells were counted in 5 consecutive non-overlapping high-power fields (HPF) 400x magnification (0.237 mm^2^/field), using an Olympus BX51 microscope (Olympus, Tokyo, Japan). The results were expressed as the mean of NKp46+/mm^2^ out of 5 regions of interest. Immunostaining and scoring were evaluated by 4 independent observers (C.D., G.B., E.L. and A.A.) blinded to patients’ clinical characteristics.

### Cytokine assay

IFN-γ was measured by ELISA on the culture supernatant collected on day 5 from suppression experiments. Cytokine concentration was assessed by Human IFN-gamma Instant ELISA (Bender MedSystems). Samples were acquired by LB 940 Multimode Reader Mithras (Berthold Technologies).

### Statistical analysis

Statistical analysis were performed using the MedCalc 9.3.7.0 and Excel software. Unpaired Student t test or non-parametrical Mann-Whitney test was conducted. Statistical tests of hypotheses was two-sided with *p* < 0.05 taken as the criterion for statistical significance.

## Results

### RCC-VHL mutations were detected in 47% of RCC patients

Clinical pathological features of patients are summarized in Table 1. The VHL status was assessed in 55 primary renal cancers by direct sequencing. As shown in Tables 1 and 2, VHL was mutated in 26/55 (47%) cases, with missense mutations in 7/26 (27%), nonsense in 3/26 (12%), splice site in 2/26 (7%), frameshift deletions in 11/26 (42%) and frameshift insertions in 3/26 (12%) cases. The mutations exon prevalence was: exon 1, 50%; exon 2, 30.7%; and exon 3, 19.3%.

**Table 1:** Patient characteristics (*N* = 55)

Age, *n* (%)	
< 65 years	26 (47)
≥ 65 years	29 (53)
Mean age ± SD, *yr*	63 ± 11
Gender, *n*	
Male	33
Female	22
Histological variant, *n*	
Clear Cell	41
Papillar	4
Cromophobe	5
Missing	5
VHL mutational status, *n*	
WT	29
MUT	26
Pathological stage, *n*	
T1	28
T2	9
T3	12
Missing	6
Furhman grade, *n*	
I	6
II	16
III	16
IV	2
Missing	15

**Table 2:** VHL mutational status in RCC patients

Exon	Mutation	Mutation type
3	c. 565C > T; p.E189X	Nonsense
3	c.481 C > T; p.R161X	Nonsense
1	c.203C > A; p.S68X	Nonsense
2	c.345_349delCCTTT; p.G114 fs*15	Frameshift
1	c.162 insG; p. M54 fs*12	Frameshift
2	c.450delT; p.N150 fs*9	Frameshift
1	c. 203_209delCGCGCGA; p. S68 fs*89	Frameshift
1	c.215_222delCCAGGTC; p. E70fs*57	Frameshift
3	c.502_505delAGCC; p. S168 fs*1	Frameshift
2	c.391_394delAACC; p.N131 fs*26	Frameshift
3	c.638_639insT; p. D213fs*2	Frameshift
1	c.199delA; p.N67Tfs*97	Frameshift
1	c.306_314delGCCTGGCA p.P102Pfs*27	Frameshift
2	c.411_413 insGT; p. V137Cfs*22	Frameshift
1	c.339_340delAG; p.G114 fs*17	Frameshift
2	c.444delT; p.F148 fs*11	Frameshift
1	c.1101delA; p.V87 fs*47	Frameshift
1	c.340G > T; p.G114C	Missense
1	c.234 T > G; p.N78 K	Missense
3	c.488 T > A; p.L163H	Missense
1	c.240 T > A; p.S80R	Missense
1	c.506C > T; p. L169P	Missense
2	c.602 T > C; p.L201R	Missense
1	c.194C > T p.S65 L	Missense
2	c.IVS2 + 2 T > C	Splicing
2	c.IVS1–1 G > C	Splicing

### VHL-MUT-RCC patients whole blood cytotoxicity was more efficient toward A498-VHL-MUT than CAKI-1-VHL-WT cells

**Fig. 1:**
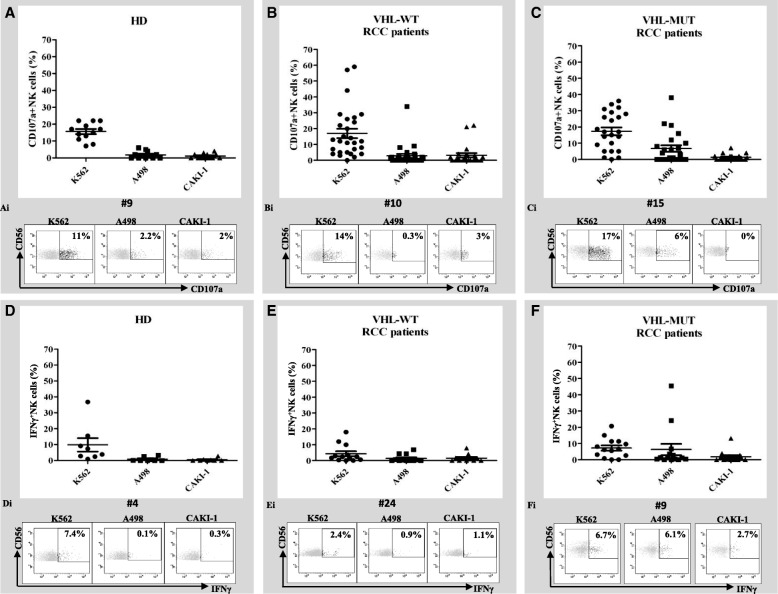
VHL-MUT-RCC patients display higher NK cytotoxicity toward A498, VHL-MUT RCC cell line. NK cell function was evaluated in whole blood through CD107a cell-surface expression and production of IFN-γ in response to ex vivo stimulation with K562, A498 and CAKI-1 cells. Degranulation (CD107a; **A, B, C**) and cytokine production (IFN-γ; **D, E, F**) were evaluated after gating on CD3^−^CD56^+^ cells. Percent of degranulation was obtained subtracting the percent of degranulation occurring in the absence of target cells. CD107a^+^NK cells in 12 HD (**A**), 28 VHL-WT (**B**) and 23 VHL-MUT (**C**) RCC patients versus K562, CAKI-1 and A498 renal cell lines. Representative profiles of the percentages of CD107a^+^NK cells in HD (**Ai**) VHL-WT (**Bi**) and VHL-MUT (**Ci**) RCC patients. IFN-γ production was assayed by intracellular cytokine staining in 8 HD (**D**), 13 VHL-WT (**E**) and 14 VHL-MUT (**F**) RCC patients versus K562, CAKI-1 and A498 renal cell lines. Representative profiles of the percentages of IFN-γ^+^ NK cells in HD (**Di**) VHL-WT (**Ei**) and VHL-MUT (**Fi**) RCC patients. The results are presented mean ± SEM. Statistical significance was calculated by unpaired Student t test (*p* < 0.05)

IL-2 activated whole-blood-NK derived cytotoxicity (as CD107a externalization and intracellular IFNγ production) was evaluated in 51 available RCC patients (28 VHL-WT and 23 VHL-MUT) and 12 healthy donors (HD). IL-2 activated whole-blood samples were co-cultured with target cells K562, A498-VHL-MUT (containing a 4-nt deletion at nt 639–642 in VHL exon 2) [26] and CAKI-1-VHL-WT cell lines. As reported in Additional file 2: Figure S1 the target cells K562 are MHC-I negative, while A498 and CAKI-1 cell lines express high levels of MHC-I molecules (81 and 86.2% respectively). In Fig. 1 NKs from HD (A) and RCC patients, either VHL-WT (B) or –MUT (C), displayed comparable externalization of CD107a against K562 cells (average CD107a^+^NK cells: 15 ± 0.02% vs 15 ± 3% vs 17 ± 2.5% respectively); absence of CD107a^+^NK cells activity was detected evaluating HD blood samples after incubation with human RCC lines (Fig. 1A). Comparable CD107a^+^NK cells exposure was observed in VHL-WT-RCC patients after stimulation with human renal cells, A498 and CAKI-1 (average CD107a^+^NK cells: 3 ± 1.3% vs 3 ± 1% respectively, *p* = 0.84, Fig. 1B). Interestingly, high percentage of NK cells expressing CD107a was observed for blood derived from VHL-MUT-RCC patients against A498-VHL-MUT cells and not versus CAKI-1-VHL-WT cells (average CD107a^+^NK: 7 ± 2% vs 1 ± 0.41% respectively, *p* = 0.015, Fig. 1C). Similar results were obtained when IL-2 activated whole blood samples from VHL-MUT-RCC patients (*n* = 5) were evaluated toward another VHL-MUT RCC cell line, 786-O cells (MHC-I expressing cells, Additional file 2: Figures S1-S2A) carrying a single mutation in VHL exon 1 (1-nt deletion at nt 523) [26] different from A498 cells. Low expression of CD107a^+^NK cells was observed incubating RCC-VHL-WT blood samples (*n* = 9) with 786-O (Additional file 2: Figure S2B). The higher percent of CD107a^+^NK induced in VHL-MUT patients by the culture with A498 and 786-O VHL-MUT cell lines suggest that RCC-VHL mutated tumors displayed higher susceptibility to NK lysis .

**Fig. 2:**
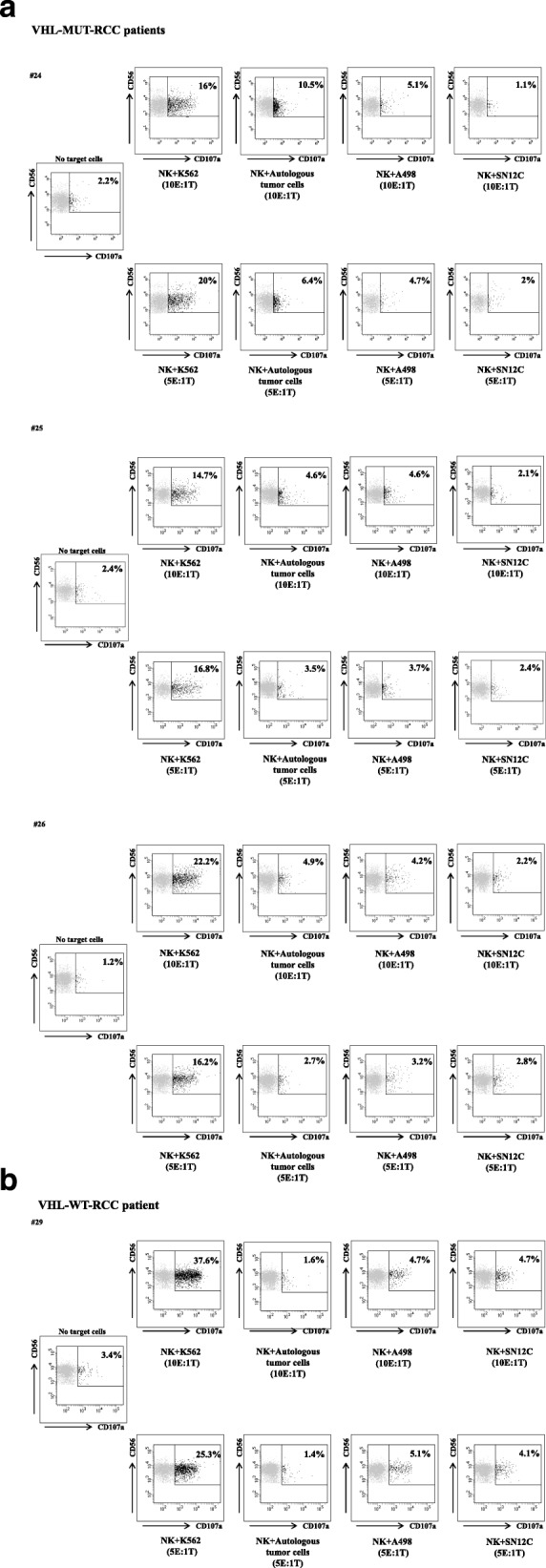
Isolated NKs from VHL-MUT-RCC patients show higher cytolytic capacity against autologous tumor cells. **a**-**b** Flow cytometry plots represent the percent NK cells isolated from 4 RCC patients (#24, #25, #26 VHL-MUT and #29 VHL-WT patients) that express CD107a following no stimulation (NK no target cells) and stimulation with K562, autologous tumor cells, A498 VHL-MUT and SN12C VHL-WT cell lines. Isolated NK cells were activated overnight with IL-2 (100 U/mL). Assays were performed at the indicated effector-target ratio (10E:1 T; 5E:1 T)

Clinical-pathological features and the CD107a assay results from single patient were shown in Additional file 3: Table S2 (VHL-MUT-RCC patients) and Additional file 4: Table S3 (VHL-WT-RCC patients). NK derived-IFN-γ was evaluated in 8 HD (Fig. 1D) versus 13 VHL-WT (E) and 14 VHL-MUT (F) RCC patients in the presence of target cells (K562, A498 and CAKI-1). The highest IFN-γ production was registered with VHL-MUT blood versus A498 cells as compared to CAKI-1 cells (average IFN-γ^+^NK: 6.26 ± 3.4% vs 1.78 ± 0.9% respectively; *p* = 0.23 Fig. 1F). In addition autologous NK cell degranulation was evaluated in the presence of MUT-VHL tumors. Patients derived (1 VHL-WT, 3 VHL-MUT)-IL-2 activated NK cells were cultured in the presence of RCC cell lines or freshly isolated autologous tumor cells. As shown in Fig. 2, NKs derived from VHL-MUT patients (#24, #25, #26) displayed higher CD107a exposure versus RCC autologous tumor cells or A498-VHL-MUT cells as compared to SN12C-VHL-WT cells (average CD107a^+^NK: 4.7 and 2.7% vs 0.3% respectively at 10E:1 T ratio). Conversely, NKs derived from VHL-WT patient (#29) did not significantly increase CD107a exposure either toward RCC autologous tumor cells than toward SN12C or A498 cell lines. Thus we can speculate that NKs from VHL-MUT-RCC patients are more efficient toward human renal cancer VHL-MUT cells.

### VHL-MUT-RCC are NKp46^+^cells infiltrated and express NK activator receptors

**Fig. 3:**
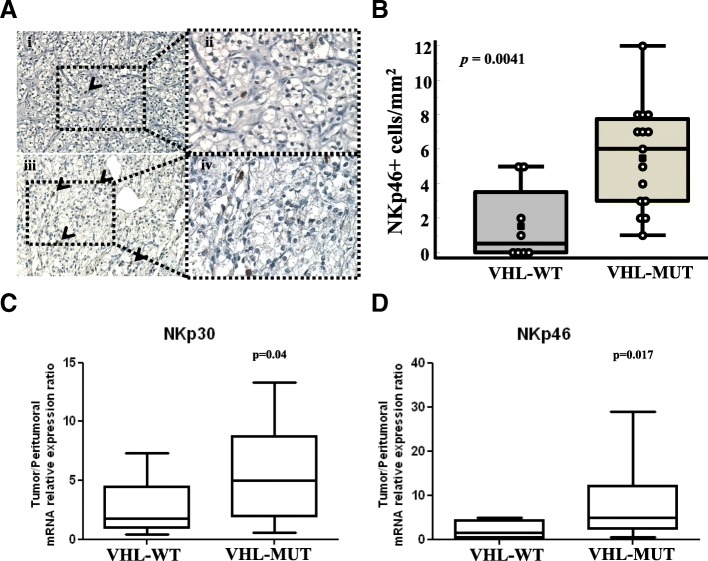
Higher NKp46^+^ cells infiltration and expression of NKp30 and NKp46 in VHL-MUT-RCCs. **A** Representative tissue staining in VHL-WT (**Ai-Aii**) and VHL-MUT-RCC patients (**Aiii-Aiv**) (Magnification 200X detail 400x). **B** NKp46^+^ cell densities [NKp46+ cells/mm^**2**^] in RCC patients grouped in VHL-WT (*n* = 8) and VHL-MUT (*n* = 15). The results are presented as mean ± SEM; *p* = 0.0041, Mann–Whitney test. **C-D** mRNA expression of NKp30 and NKp46 in 17 VHL-WT and 17 VHL-MUT RCC patients. Transcript levels are presented as mean ± SEM. Statistical significance was calculated by unpaired Student t test (*p* < 0.05)

With the intent to correlate peripheral and tumoral NK status, NK cell infiltration was analyzed on paraffin-embedded primary RCC tissues by immunohistochemical staining using the NK cell–specific marker NKp46. 23 RCC-patients, 15/23 (65.2%) VHL-MUT and 8/23 (34.8%) VHL-WT were evaluated for the expression of NKp46^+^ cells. Although RCCs generally display low NK cell infiltration [27], NKp46 was expressed in the cytosol and on cell surface in lymphocytes (Fig. 3A). In VHL-WT-RCC the NKp46^+^ cells ranged from 0 to 5 per mm^2^ (median 0.5 cells/mm^2^), (4/8 VHL-WT-RCC were totally negative for NKp46). In 15 VHL-MUT-RCCs NKp46^+^ cells ranged from 1 to 12 per mm^2^ (median 6 cells/mm^2^); a significant higher number of NKp46^+^cells was detected in VHL-MUT-RCCs as compared to VHL-WT-RCCs (average NKp46^+^cells: 5.553 ± 0.78 vs 1.625 ± 0.78, *p* = 0.0041, Mann–Whitney test, Fig. 3B).

NKs function is regulated by activating receptors other than NKp46 that include NKp30, NKp44, C-type lectin receptors NKG2D, CD94/NKG2C, FcγRIIIa (CD16), activator KIRs, DNAX accessory molecule-1 (DNAM-1, CD226), and costimulatory receptors such as NCAM-1, and the main NK cell-activating ligands on A498 and CAKI-1 cells was recently reported [27–29]. The expression of NKp30, NKp44, NKp46, NKG2D, DNAM-1, NCAM-1 and FcγRIIIa was evaluated by RT-PCR analysis in VHL-WT (*n* = 17) and VHL-MUT (n = 17) RCC samples. A significant high expression of NKp30 and NKp46 was detected in VHL-MUT-RCC as compared to VHL-WT-RCC samples (*p* = 0.04 and *p* = 0.017, respectively, Fig. 3C and D) and NKp44 and DNAM-1 were slightly overexpressed in VHL-MUT compared to VHL-WT patients. Conversely expression of NCAM-1, FcγRIIIa and NKG2D were comparable in the two groups (Additional file 2: Figure S3). In addition, NKp30, NKp44, NKp46 and FcγRIIIIa mRNA were highly expressed in VHL-MUT tumors as compared to surrounding unaffected tissue (Additional file 2: Figure S4A) while in the VHL-WT tumors no significant difference was retrieved (Additional file 2: Figure S4B). Thus VHL-MUT tumors were characterized by high density of NKp46^+^ cells infiltration and high expression of NK activating genes such as NKp30, NKp46, NKp44 and DNAM-1.

### VHL-MUT-RCC tumors display less functional Tregs

**Fig. 4:**
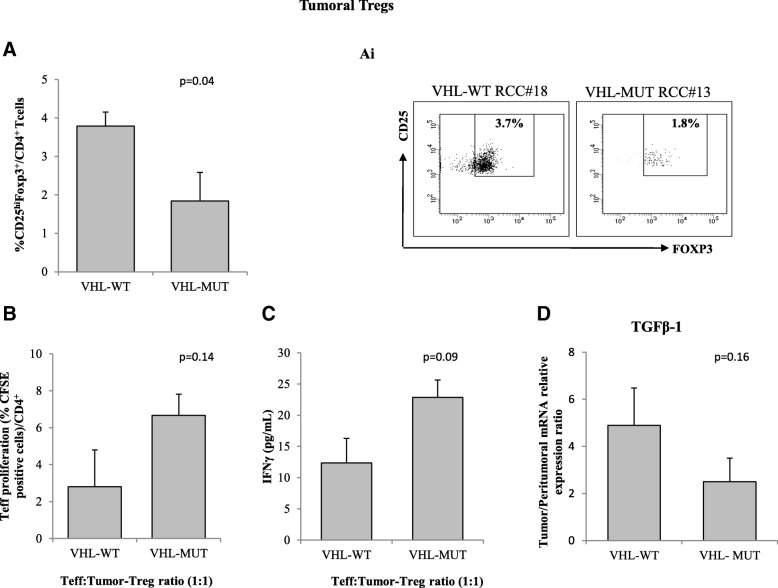
Tumor-Tregs from VHL-WT-RCC patients are more suppressive than tumor-Tregs from VHL-MUT-RCC patients. **A** Percentage of tumor CD4^+^CD25^hi^Foxp3^+^cells (13 VHL-WT-RCCs vs 13 VHL-MUT-RCCs, *p* = 0.04). **(Ai)** Representative plots of Tregs from 1 VHL-WT-RCCs vs 1 VHL-MUT-RCCs. **B** Autologous CFSE-labeled CD4^+^CD25^−^ T cells were co-cultured with CD4^+^CD25^+^ isolated from tumor of 6 VHL-WT and 4 VHL-MUT RCC patients. After 5 days of stimulation with Dynabeads Human T-Activator CD3/CD28, CFSE^+^CD4^+^ T cells were analyzed for their proliferation by CFSE dilution. **C** IFN-γ-tumor-Treg dependent ELISA from 3 VHL-WT vs 3 VHL-MUT RCC patients. **D** Measurement of TGF-β1 mRNA expression in 6 VHL-WT vs 6 VHL-MUT RCC patients by real-time PCR in tumor and corresponding peritumoral tissues. Relative mRNA expression was calculated according to 2^-ΔΔCt^ method. The results are presented mean ± SEM. Statistical significance was calculated by unpaired Student t test (*p* < 0.05)

Since Tregs are supposed to suppress NK activity tumoral, Tregs (tTreg) identified as CD4^+^CD25^hi^Foxp3^+^ cells, were evaluated in 26 RCC patients (13 VHL-WT and 13 VHL-MUT). tTregs were significantly lower in VHL-MUT-RCC as compared to VHL-WT-RCC tumors (1.84 ± 0.36% vs 3.79 ± 0.74%; p = 0.04, Fig. 4A). Treg function was evaluated ex vivo on patients T effectors (Teff) proliferation. Tregs isolated from VHL-MUT tumors were less efficient in inhibiting T-effectors proliferation (VHL-MUT: 6.7 ± 3.9% vs VHL-WT: 2.8 ± 1.1%, *p* = 0.14 Fig. 4B) as confirmed by increased IFN-γ production in co-colture of Tregs:Teff isolated from VHL-MUT-RCC patients (22.8 ± 2.8 vs 12.36 ± 3.9 pg/mL, *p* = 0.09 Fig. 4C). Few patients were also evaluated for TGF-β1 mRNA (6 VHL-WT/6 VHL-MUT peritumoral and tumoral tissues). Consistent with a reduction of Tregs function, TGF-β1 expression, though not significant, was lower in VHL-MUT RCC as compared to VHL-WT-RCC (2.5 ± 1.0 vs 4.9 ± 1.15, *p* = 0.16 Fig. 4D).

## Discussion

In the present study RCC-VHL mutational status was correlated to patients NK cytotoxicity through ex vivo evaluation toward human renal cancer cell lines A498 VHL-MUT and CAKI-1-VHL-WT. In 51 RCC patients (23 VHL-MUT and 28 VHL-WT) enhanced NK cell cytotoxicity, as CD107a expression in whole blood, was reported versus VHL-MUT A498 cells in VHL-MUT RCC patients. Moreover, in an autologous setting, IL-2 activated NKs isolated from RCC-VHL-MUT patients more efficiently degranulated toward RCC autologous tumor cells or A498 VHL-MUT cells as compared to SN12C (RCC-VHL-WT) cell line. Higher NK susceptibility was previously reported to correlate with decreased MHC Class I expression in cancer cells [30]. Herein it was shown that A498 (containing a 4-nt deletion at nt 639–642 in VHL exon 2) and 786-O (containing a 1-nt deletion at nt 523 in VHL exon 1), express high level of classical MHC-I molecules not significantly different from CAKI-1, VHL-WT (A498: 81%; 786-O: 92.7% and CAKI-1: 86.2%) suggesting that tested MHC-I molecules are not mainly involved in the different NK sensitivity. It is noteworthy that A498 and 786–0 cell lines, although carrying a MUT-VHL, display phenotypical and molecular differences. 786–0 appearing poorly differentiated with sarcomatoid features while A498 displays epithelial cells with clear cytoplasm. Moreover 786-O carries (VHL−/− and PTEN−/−), A498 (VHL−/− and PTEN+/+) and CAKI-1 carries (VHL+/+ and PTEN+/+) [31, 32]. These differences might accomplish for different phenotypes affecting NK sensitivity [15].

Nowadays the effects of VHL mutations on NK activity is controversial. Previous evidence demonstrated that restoration of wild type VHL in human renal cancer cells decreased their NK susceptibility [33] while VHL mutations increased resistance to NK-mediated lysis through EPAS1/HIF-2α stabilization [16, 34]. NK cells induced a contact-dependent autophagy in ccRCC cells that was mediated by the HIF-2a targeted, inositol triphosphate receptor1 (ITPR1) in tumor cells. Blocking ITPR1 expression in ccRCC cells inhibited NK cell-induced autophagy and suppressed ccRCC resistance to NK cells [35].

In our study blood derived from patients carrying RCC-VHL-MUT tumors display better NK susceptibility toward human renal cancer cells VHL-MUT suggesting an improvement in the innate immune response toward RCC.

To further correlate peripheral and tumoral NK status, NK cell infiltration was analyzed through the evaluation of NKp46^+^ cells and the NKs activating receptors NKp30, NKp44, NKp46, NKG2D, DNAM-1, NCAM-1 and FcγRIIIa. NK cell-activating ligands expression on A498 and CAKI-1 cells was recently reported [27–29]. Although a significant increase in NKG2D+ cells was not detected in our tested cells, A498 and CAKI-1 express high levels of DNAM-1 ligand PVR (94.8 and 99.1% respectively, Additional file 2: Figure S5). Previous authors stated that VHL-mutational status may not affect the expression of ligands for NKG2D and DNAM-1 activating NK receptors [33]. Higher infiltration of NKp46^+^ cells and increased expression of NKp30 and NKp46 [36–38] were detected in VHL-MUT-RCC compared to VHL-WT-RCC. Although the NKp46 receptor demonstrates a high degree of lineage-specificity, being expressed almost exclusively in natural killer cells [39] the presence of a rare NKp46^+^CD3^+^ population was reported. These include rare subsets of T cells (αβ and δγ) in humans and mice and a subset of group 3 innate lymphoid cells (ILCs) (NCR^+^ ILC3) [36, 40]. Also DNAM1 or NKG2D may be expressed by T cells [36, 40, 41]. This is a limit of the present study. However the concomitant expression of NKp46 plus NKp30, NKp44 and DNAM-1 strongly suggest the hypothesis that NKs are involved.

To better define the role of NK cells in RCC tumors, Tregs and NKs are currently evaluated within the study REVOLUTION aimed at identify biomarkers of the checkpoint inhibitor nivolumab efficacy in metastatic renal cancer patients. Preliminary evidence showed that while the absolute value of CD3^−^CD56^dim^ was not affected by nivolumab treatment NK cytotoxicity correlated with the responsiveness to treatment (Proceedings AACR 2017: Abstract #580).

Tregs suppress NK cell effector functions [42] impairing TGF-β pathway which, among other effects, induces downregulation of the activating NK cell receptor NKG2D [43]. Herein we report high infiltration of NKp46^+^ cells and reduced CD4^+^CD25^hi^Foxp3^+^ T cells in VHL-MUT-RCC tumors, although the reduction of TGF-β levels did not correspond to increased expression of NKG2D gene in VHL-MUT-RCC tumors. As in our results, Shen showed no significant correlation between peripheral Tregs and NKG2D expression in colorectal cancer patients NK cells [44]. Since VHL mutations induces PD-L1 expression in RCC, NK function may be a crucial element in nivolumab sensitivity [45]. NKs isolated from renal carcinoma patients expressed PD-1 on their surface and engagement of PD-1 signaling reduced their cytolytic potential. Treatment of patient-derived PD-1^+^ NK cells with an anti-PD-1 antibody (pidilizumab, CT-011) increased NK killing of autologous cancer cells in vitro [46]. NK cells also engage antibody-dependent cell mediated cytotoxicity (ADCC) and ex vivo treatment with the anti-CTLA4 ipilimumab enhances cetuximab-mediated ADCC targeting CTLA-4^+^ Treg in head and neck squamous cell cancer (SCCHN)[47, 48]. Nowadays the immune checkpoint targeting agents nivolumab and ipilimumab represent a clinical standard for mRCC. Nevertheless tyrosine kinase inhibitors (TKI) still play a central role in metastatic RCC treament [49] and modify the tumor immune sensitivity. TKIs affect the frequency and composition of tumor immune cell subpopulations including Tregs, myeloid-derived suppressor cells as well as T and NK cells [50–52]. Sorafenib significantly reduce NKs activity against the RCC cell lines A498, ACHN and CAKI-2 [53] while axitinib promotes NK cell recognition and degranulation toward A498 RCC cells in a ROS-dependent manner [28].

## Conclusions

VHL tumoral mutations improve the NKs effectiveness in RCC patients and thus need to be considered in the evaluation of TKIs or immune based therapies. Moreover therapeutic strategies designed to target NK cells could be beneficial in VHL-mutated-RCCs alone or in association with immune checkpoints inhibitors.

## Additional files

Additional file 1: **Table S1**: Primer sequences for SYBR Green RT-qPCR. (PPTX 68 kb)

Additional file 2: **Figure S1.** MHC Class-I profile in K562, CAKI-1, SN12C, A498 and 786-O cells. The expression of MHC-I was evaluated in RCC target cells by flow cytometry (FITC-conjugated major histocompatibility complex (MHC) class I–specific antibody (IgG2a, W6/32, CBL139F, Cymbus Biotech, Hants, UK). **Figure S2.** NKs from VHL-MUT-RCC patients display higher cytotoxicity toward human renal cancer cells VHL mutated A498 and 786-O. NK cell function was evaluated through CD107a cell-surface expression in response to ex vivo stimulation with K562, CAKI-1 (VHL-WT), A498 and 786-O (VHL-MUT) cells. Degranulation (CD107a) was evaluated after gating on CD3^-^CD56^+^ cells. CD107a^+^NK cells in 5 VHL-MUT (A) and 9 VHL-WT (B) RCC patients versus K562, CAKI-1, A498 and 786-O renal cell lines. **Figure S3.** NCAM-1, DNAM-1, FcγRIIIa, NKp44 and NKG2D are slightly overexpressed in VHL-MUT RCC tumors. RNA from 34 RCC tumors (17 VHL-WT and 17 VHL-MUT). Transcript levels are presented as mean±SEM. Statistical significance was calculated by unpaired Student t test (p < 0.05 ). **Figure S4.** NCAM-1, DNAM-1, FcγRIIIa, NKp30, NKp46, NKp44, NKG2D expression are upregulated in VHL-MUT tumors as compared to VHL-WT tumors. 17 VHL-MUT (A) and 17 VHL-WT (B). RT-PCR was performed on total RNA isolated from 34 tumors and relative peritumoral tissues (17 VHL-WT and 17 VHL-MUT). Relative gene expression levels were normalized to GUSB. Statistical significance was calculated by unpaired Student t test (p < 0.05). **Figure S5.** Expression of DNAM-1 ligand (PVR) in CAKI-1 and A498 cell lines. The expression of PVR (CD155) was evaluated in RCC target cells by flow cytometry (PE anti-human CD155/PVR, ( clone SKIL.4, Biolegend, Cat No 337609). (ZIP 561 kb)

Additional file 3: **Table S2.** Detailed characteristics of 23 VHL-MUT-RCC patients. (PPTX 71 kb)

Additional file 4: **Table S3.** Detailed characteristics of 28 VHL-WT-RCC patients. (PPTX 71 kb)

## Abbreviations

ADCC: Antibody-dependent cell mediated cytotoxicity
ccRCC: Clear cell renal cell carcinoma
CFSE: Carboxyfluorescein diacetate succinimidyl ester
CLRs: C-type lectin receptors
HD: Healthy donors
HIFs: Hypoxia-inducible factors
ITRI1: Inositol triphosphate receptor1
KIRs: Killer cell immunoglobulin-like receptors
LAMP-1 (CD107a): Lysosome-associated membrane protein
NCRs: Natural cytotoxicity receptors
NKs: Natural killer cells
PB: Peripheral blood
PT: Peritumoral tissue
RCCs: Renal cell carcinomas
SCCHN: Head and neck squamous cell cancer
Teff: T effector cells
TGF-β: Transforming growth factor-beta
TKI: Tyrosine kinase inhibitor
Tregs: T regulatory cells
TT: Tumor tissue
VHL: Von Hippel-Lindau
VHL-MUT: Von Hippel-Lindau mutated
VHL-WT: Von Hippel-Lindau wild-type
