# Supplementary material for: Mutated Von Hippel-Lindau-renal cell carcinoma (RCC) promotes patients specific natural killer (NK) cytotoxicity
Source: J Exp Clin Cancer Res. 2018 Dec 4;37:297. doi: 10.1186/s13046-018-0952-7 (PMC6278085; doi:10.1186/s13046-018-0952-7)
Supplement: Supplementary file 1 — Table S1: Primer sequences for SYBR Green RT-qPCR. (PPTX 68 kb) [file 13046_2018_952_MOESM1_ESM.pptx]

## Slide 1
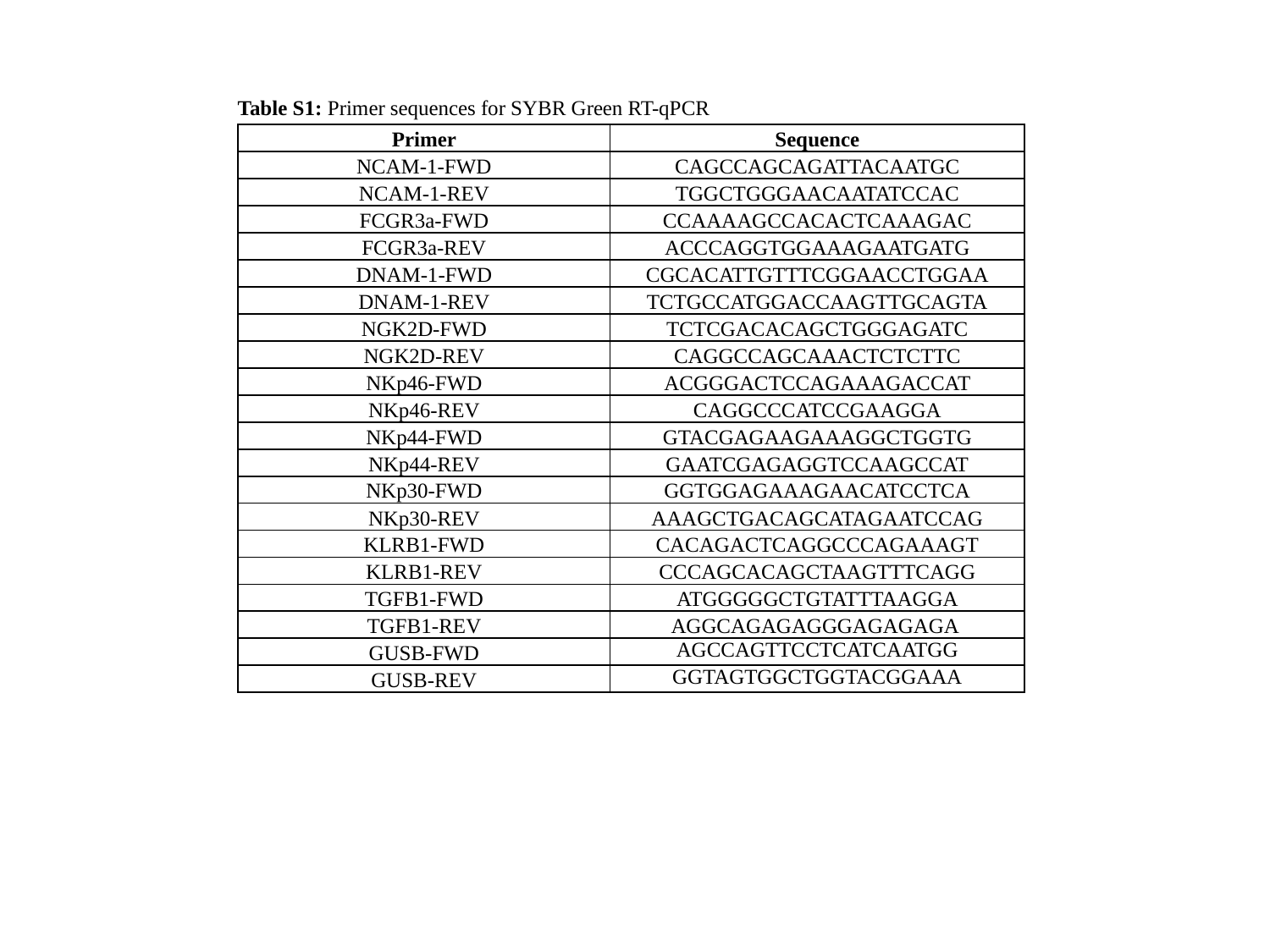

Table S1: Primer sequences for SYBR Green RT-qPCR
| Primer | Sequence |
| --- | --- |
| NCAM-1-FWD | CAGCCAGCAGATTACAATGC |
| NCAM-1-REV | TGGCTGGGAACAATATCCAC |
| FCGR3a-FWD | CCAAAAGCCACACTCAAAGAC |
| FCGR3a-REV | ACCCAGGTGGAAAGAATGATG |
| DNAM-1-FWD | CGCACATTGTTTCGGAACCTGGAA |
| DNAM-1-REV | TCTGCCATGGACCAAGTTGCAGTA |
| NGK2D-FWD | TCTCGACACAGCTGGGAGATC |
| NGK2D-REV | CAGGCCAGCAAACTCTCTTC |
| NKp46-FWD | ACGGGACTCCAGAAAGACCAT |
| NKp46-REV | CAGGCCCATCCGAAGGA |
| NKp44-FWD | GTACGAGAAGAAAGGCTGGTG |
| NKp44-REV | GAATCGAGAGGTCCAAGCCAT |
| NKp30-FWD | GGTGGAGAAAGAACATCCTCA |
| NKp30-REV | AAAGCTGACAGCATAGAATCCAG |
| KLRB1-FWD | CACAGACTCAGGCCCAGAAAGT |
| KLRB1-REV | CCCAGCACAGCTAAGTTTCAGG |
| TGFB1-FWD | ATGGGGGCTGTATTTAAGGA |
| TGFB1-REV | AGGCAGAGAGGGAGAGAGA |
| GUSB-FWD | AGCCAGTTCCTCATCAATGG |
| GUSB-REV | GGTAGTGGCTGGTACGGAAA |
