# Supplementary material for: Mutated Von Hippel-Lindau-renal cell carcinoma (RCC) promotes patients specific natural killer (NK) cytotoxicity
Source: J Exp Clin Cancer Res. 2018 Dec 4;37:297. doi: 10.1186/s13046-018-0952-7 (PMC6278085; doi:10.1186/s13046-018-0952-7)
Supplement: Supplementary file 2 — Figure S1. MHC Class-I profile in K562, CAKI-1, SN12C, A498 and 786-O cells. The expression of MHC-I was evaluated in RCC target cells by flow cytometry (FITC-conjugated major histocompatibility complex (MHC) class I–specific antibody (IgG2a, W6/32, CBL139F, Cymbus Biotech, Hants, UK). Figure S2. NKs from VHL-MUT-RCC patients display higher cytotoxicity toward human renal cancer cells VHL mutated A498 and 786-O. NK cell function was evaluated through CD107a cell-surface expression in response to ex vivo stimulation with K562, CAKI-1 (VHL-WT), A498 and 786-O (VHL-MUT) cells. Degranulation (CD107a) was evaluated after gating on CD3-CD56+ cells. CD107a+NK cells in 5 VHL-MUT (A) and 9 VHL-WT (B) RCC patients versus K562, CAKI-1, A498 and 786-O renal cell lines. Figure S3. NCAM-1, DNAM-1, FcγRIIIa, NKp44 and NKG2D are slightly overexpressed in VHL-MUT RCC tumors. RNA from 34 RCC tumors (17 VHL-WT and 17 VHL-MUT). Transcript levels are presented as mean±SEM. Statistical significance was calculated by unpaired Student t test (p < 0.05 ). Figure S4. NCAM-1, DNAM-1, FcγRIIIa, NKp30, NKp46, NKp44, NKG2D expression are upregulated in VHL-MUT tumors as compared to VHL-WT tumors. 17 VHL-MUT (A) and 17 VHL-WT (B). RT-PCR was performed on total RNA isolated from 34 tumors and relative peritumoral tissues (17 VHL-WT and 17 VHL-MUT). Relative gene expression levels were normalized to GUSB. Statistical significance was calculated by unpaired Student t test (p < 0.05). Figure S5. Expression of DNAM-1 ligand (PVR) in CAKI-1 and A498 cell lines. The expression of PVR (CD155) was evaluated in RCC target cells by flow cytometry (PE anti-human CD155/PVR, ( clone SKIL.4, Biolegend, Cat No 337609). (ZIP 561 kb) [file 13046_2018_952_MOESM2_ESM.zip › figure S3.pptx]

## Slide 1
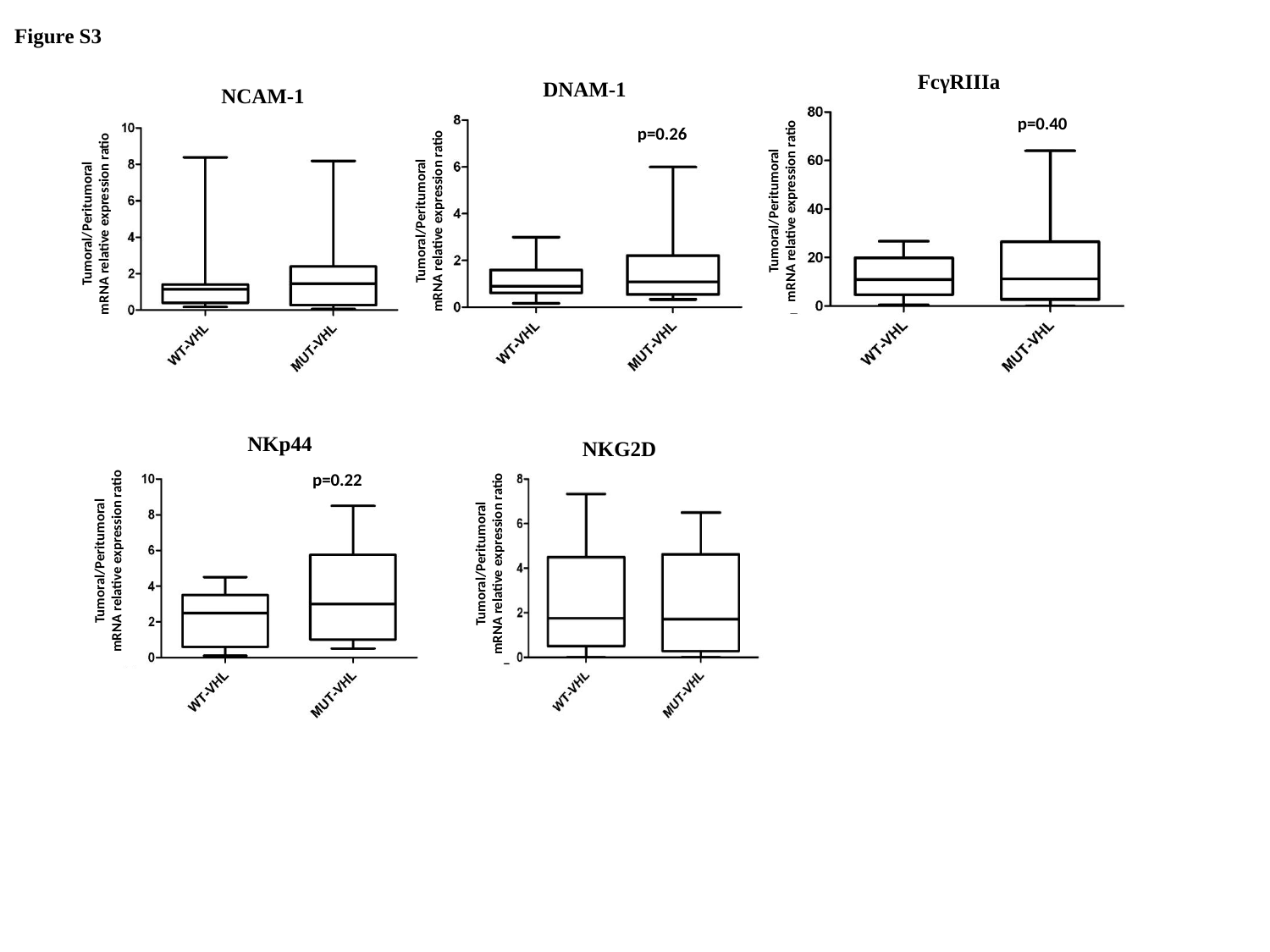

Figure S3
Tumoral/Peritumoral
mRNA relative expression ratio
p=0.40
FcγRIIIa
Tumoral/Peritumoral
mRNA relative expression ratio
p=0.26
DNAM-1
Tumoral/Peritumoral
mRNA relative expression ratio
NCAM-1
p=0.75
Tumoral/Peritumoral
mRNA relative expression ratio
p=0.22
NKp44
Tumoral/Peritumoral
mRNA relative expression ratio
NKG2D
p=0.62
