# Supplementary material for: Mutated Von Hippel-Lindau-renal cell carcinoma (RCC) promotes patients specific natural killer (NK) cytotoxicity
Source: J Exp Clin Cancer Res. 2018 Dec 4;37:297. doi: 10.1186/s13046-018-0952-7 (PMC6278085; doi:10.1186/s13046-018-0952-7)
Supplement: Supplementary file 3 — Table S2. Detailed characteristics of 23 VHL-MUT-RCC patients. (PPTX 71 kb) [file 13046_2018_952_MOESM3_ESM.pptx]

## Slide 1
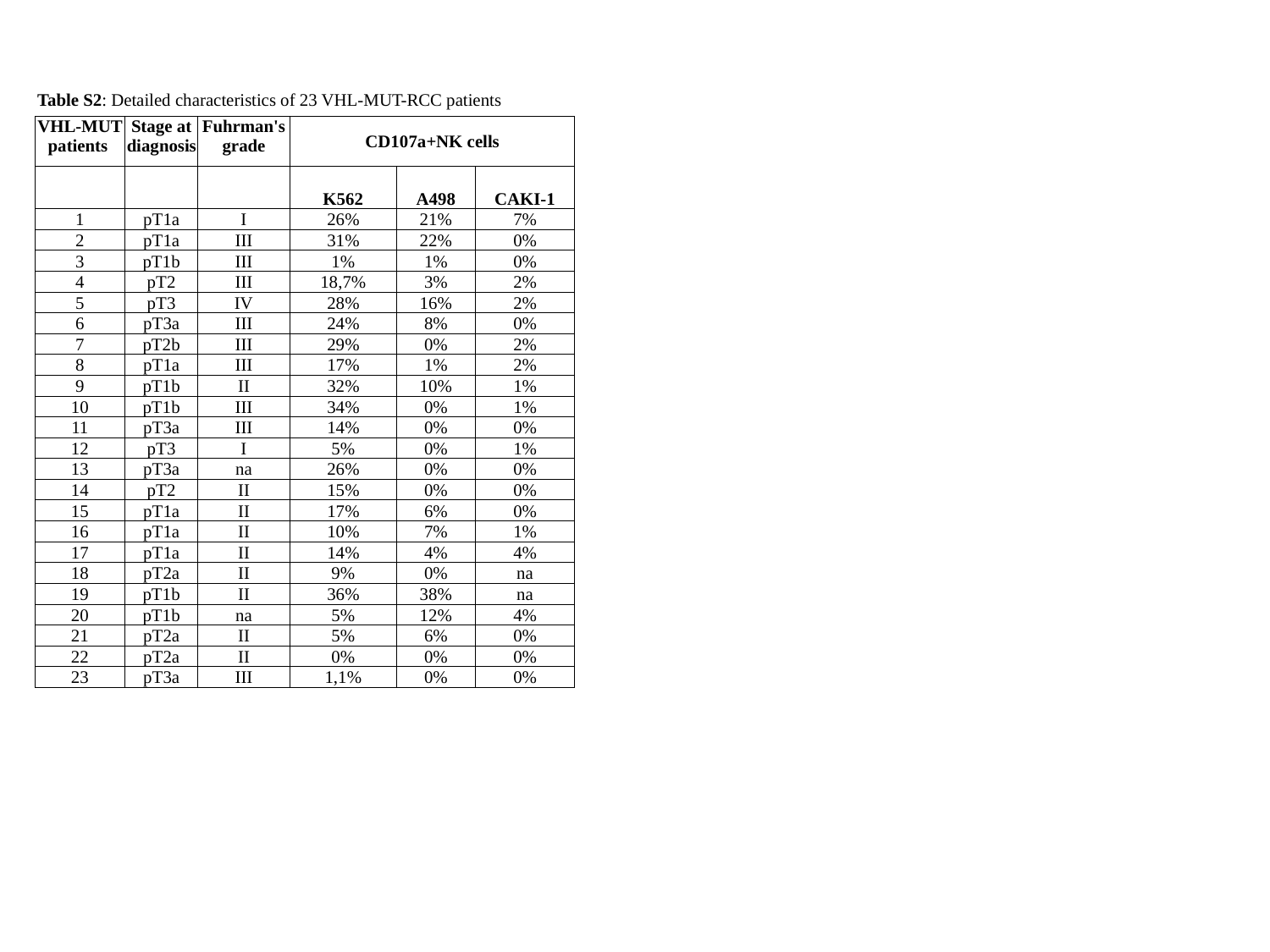

Table S2: Detailed characteristics of 23 VHL-MUT-RCC patients
| VHL-MUT patients | Stage at diagnosis | Fuhrman's grade | CD107a+NK cells | | |
| --- | --- | --- | --- | --- | --- |
| | | | K562 | A498 | CAKI-1 |
| 1 | pT1a | I | 26% | 21% | 7% |
| 2 | pT1a | III | 31% | 22% | 0% |
| 3 | pT1b | III | 1% | 1% | 0% |
| 4 | pT2 | III | 18,7% | 3% | 2% |
| 5 | pT3 | IV | 28% | 16% | 2% |
| 6 | pT3a | III | 24% | 8% | 0% |
| 7 | pT2b | III | 29% | 0% | 2% |
| 8 | pT1a | III | 17% | 1% | 2% |
| 9 | pT1b | II | 32% | 10% | 1% |
| 10 | pT1b | III | 34% | 0% | 1% |
| 11 | pT3a | III | 14% | 0% | 0% |
| 12 | pT3 | I | 5% | 0% | 1% |
| 13 | pT3a | na | 26% | 0% | 0% |
| 14 | pT2 | II | 15% | 0% | 0% |
| 15 | pT1a | II | 17% | 6% | 0% |
| 16 | pT1a | II | 10% | 7% | 1% |
| 17 | pT1a | II | 14% | 4% | 4% |
| 18 | pT2a | II | 9% | 0% | na |
| 19 | pT1b | II | 36% | 38% | na |
| 20 | pT1b | na | 5% | 12% | 4% |
| 21 | pT2a | II | 5% | 6% | 0% |
| 22 | pT2a | II | 0% | 0% | 0% |
| 23 | pT3a | III | 1,1% | 0% | 0% |
