# Supplementary material for: Mutated Von Hippel-Lindau-renal cell carcinoma (RCC) promotes patients specific natural killer (NK) cytotoxicity
Source: J Exp Clin Cancer Res. 2018 Dec 4;37:297. doi: 10.1186/s13046-018-0952-7 (PMC6278085; doi:10.1186/s13046-018-0952-7)
Supplement: Supplementary file 4 — Table S3. Detailed characteristics of 28 VHL-WT-RCC patients. (PPTX 71 kb) [file 13046_2018_952_MOESM4_ESM.pptx]

## Slide 1
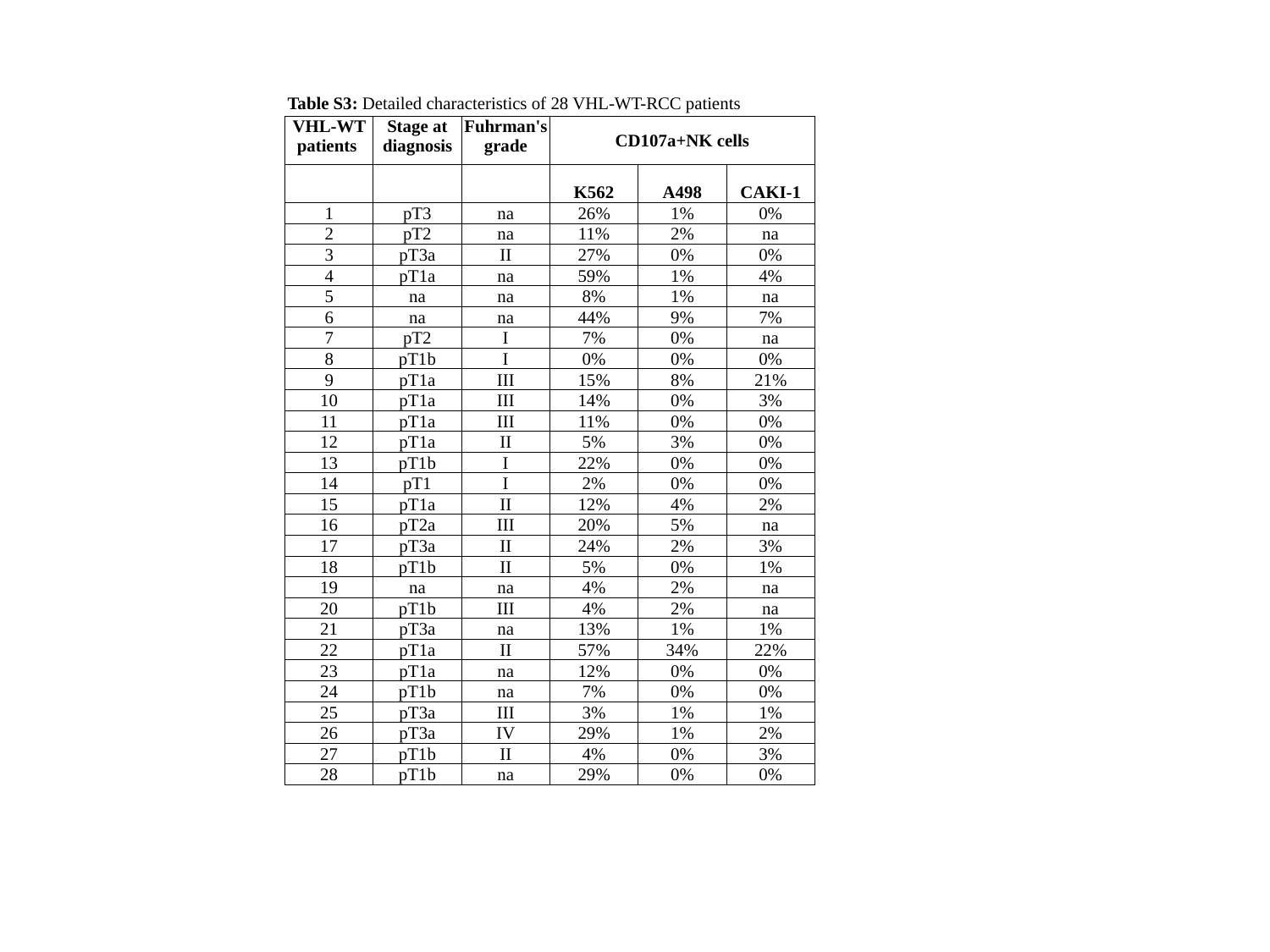

Table S3: Detailed characteristics of 28 VHL-WT-RCC patients
| VHL-WT patients | Stage at diagnosis | Fuhrman's grade | CD107a+NK cells | | |
| --- | --- | --- | --- | --- | --- |
| | | | K562 | A498 | CAKI-1 |
| 1 | pT3 | na | 26% | 1% | 0% |
| 2 | pT2 | na | 11% | 2% | na |
| 3 | pT3a | II | 27% | 0% | 0% |
| 4 | pT1a | na | 59% | 1% | 4% |
| 5 | na | na | 8% | 1% | na |
| 6 | na | na | 44% | 9% | 7% |
| 7 | pT2 | I | 7% | 0% | na |
| 8 | pT1b | I | 0% | 0% | 0% |
| 9 | pT1a | III | 15% | 8% | 21% |
| 10 | pT1a | III | 14% | 0% | 3% |
| 11 | pT1a | III | 11% | 0% | 0% |
| 12 | pT1a | II | 5% | 3% | 0% |
| 13 | pT1b | I | 22% | 0% | 0% |
| 14 | pT1 | I | 2% | 0% | 0% |
| 15 | pT1a | II | 12% | 4% | 2% |
| 16 | pT2a | III | 20% | 5% | na |
| 17 | pT3a | II | 24% | 2% | 3% |
| 18 | pT1b | II | 5% | 0% | 1% |
| 19 | na | na | 4% | 2% | na |
| 20 | pT1b | III | 4% | 2% | na |
| 21 | pT3a | na | 13% | 1% | 1% |
| 22 | pT1a | II | 57% | 34% | 22% |
| 23 | pT1a | na | 12% | 0% | 0% |
| 24 | pT1b | na | 7% | 0% | 0% |
| 25 | pT3a | III | 3% | 1% | 1% |
| 26 | pT3a | IV | 29% | 1% | 2% |
| 27 | pT1b | II | 4% | 0% | 3% |
| 28 | pT1b | na | 29% | 0% | 0% |
